# Provably Secure Mutual Authentication and Key Agreement Scheme Using PUF in Internet of Drones Deployments

**DOI:** 10.3390/s23042034

**Published:** 2023-02-10

**Authors:** Yohan Park, Daeun Ryu, Deokkyu Kwon, Youngho Park

**Affiliations:** 1 School of Computer Engineering, Keimyung University, Daegu 42601, Republic of Korea; 2 School of Electronics Engineering, Kyungpook National University, Daegu 41566, Republic of Korea

**Keywords:** AVISPA, BAN logic, Internet of Drones, mutual authentication, PUF

## Abstract

Internet of Drones (IoD), designed to coordinate the access of unmanned aerial vehicles (UAVs), is a specific application of the Internet of Things (IoT). Drones are used to control airspace and offer services such as rescue, traffic surveillance, environmental monitoring, delivery and so on. However, IoD continues to suffer from privacy and security issues. Firstly, messages are transmitted over public channels in IoD environments, which compromises data security. Further, sensitive data can also be extracted from stolen mobile devices of remote users. Moreover, drones are susceptible to physical capture and manipulation by adversaries, which are called drone capture attacks. Thus, the development of a secure and lightweight authentication scheme is essential to overcoming these security vulnerabilities, even on resource-constrained drones. In 2021, Akram et al. proposed a secure and lightweight user–drone authentication scheme for drone networks. However, we discovered that Akram et al.’s scheme is susceptible to user and drone impersonation, verification table leakage, and denial of service (DoS) attacks. Furthermore, their scheme cannot provide perfect forward secrecy. To overcome the aforementioned security vulnerabilities, we propose a secure mutual authentication and key agreement scheme between user and drone pairs. The proposed scheme utilizes physical unclonable function (PUF) to give drones uniqueness and resistance against drone stolen attacks. Moreover, the proposed scheme uses a fuzzy extractor to utilize the biometrics of users as secret parameters. We analyze the security of the proposed scheme using informal security analysis, Burrows–Abadi–Needham (BAN) logic, a Real-or-Random (RoR) model, and Automated Verification of Internet Security Protocols and Applications (AVISPA) simulation. We also compared the security features and performance of the proposed scheme and the existing related schemes. Therefore, we demonstrate that the proposed scheme is suitable for IoD environments that can provide users with secure and convenient wireless communications.

## 1. Introduction

**Figure 1:**
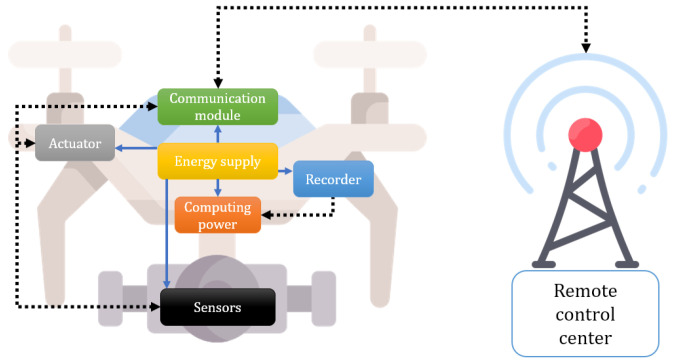
Basic structure of the drone in IoD environments.

Internet of Drones (IoD) [1], which is often referred to as an unmanned aerial vehicles (UAVs) network, is a layered network control architecture designed to coordinate the access of drones. Drones in IoD environments can perform various flight tasks by embedding various sensors, actuators, recorders, batteries, computations, and communication modules. Figure 1 shows the basic structure of a drone in IoD environments. With these modules, drones are used to control the airspace and offer services such as rescue, healthcare, traffic surveillance, environmental monitoring, delivery, and search to users [2]. The IoD architecture generally comprises remote users, a control server, and drones. Remote users query the information of drones to receive useful services. The control server is centrally located in the wireless communication flow, mediating and providing a seamless data exchange process between remote users and drones. Drones, located in their own flying zone, collect surrounding environment information and send it to users through the control center.

Although IoD environments offer useful services to users, they can suffer from several privacy and security issues [3]. Firstly, IoD environments can be vulnerable to various security attacks, such as eavesdropping, deleting, and intercepting, because all messages are transmitted via a public channel. Moreover, the mobile devices of remote users can be stolen/lost, and the sensitive stored data of these devices can threaten the whole IoD environment. Additionally, drones can be physically captured by malicious adversaries who can try to impersonate them using secret information extracted from drones using power analysis attacks. Finally, drones in IoD environments are designed to use restricted power, computation, and storage sources because the entire energy source is preferentially devoted to flying tasks. Thus, a secure and lightweight authentication scheme is necessary, considering the above security vulnerabilities and specific features of IoD environments.

In 2021, Akram et al. [4] proposed a user–drone access scheme designed to be secure and lightweight for drone networks. The authors claimed that the scheme resists user, control center, and drone impersonation attacks and provides anonymity and untraceability. However, we find that Akram et al.’s scheme is vulnerable to drone impersonation, verification table leakage, and denial of service (DoS) attacks. In addition, their scheme cannot ensure perfect forward secrecy and fails to guarantee correctness. To improve these vulnerabilities, we propose a mutual authentication and key agreement (MAKA) scheme that can provide convenient services to users with high security and efficiency for IoD environments. In the proposed scheme, we utilize biometrics [5] to resist various security attacks, such as offline guessing attacks on user devices. Moreover, we apply physical unclonable function (PUF) [6] technology to prevent cloning and physical attacks of drones using power analysis attacks. Considering real-time communication in IoD environments and the limited computation resources of user devices and drones, we only utilize hash functions and exclusive-OR operators, which are reliable in terms of computation and communication overheads.

### 1.1. Research Contributions

- We review and perform a security analysis of Akram et al.’s scheme. Then, we propose a MAKA scheme designed to ensure high security using biometrics and PUF. Hash functions and exclusive-OR operations are used for lightweight architecture, making the proposed scheme suitable for drone networks. Moreover, a fuzzy extractor and PUF are applied in the proposed scheme to enhance the security level.
- We prove the security robustness of the proposed scheme using the Automated Verification of Internet Security Protocols and Applications (AVISPA) simulation tool [7,8], Real-or-Random (RoR) model [9], and Burrows–Abadi–Needham (BAN) logic [10].
- We perform an informal analysis to ensure that the proposed scheme can provide security against various attacks, including offline password guessing, session key disclosure, verification table leakage, impersonation, and DoS attacks. Additionally, we show that the proposed scheme can achieve mutual authentication, perfect forward secrecy, untraceability, and anonymity.
- We evaluate and compare the security features, communication, and computation costs of the proposed scheme with existing authentication schemes, including Akram et al.’s scheme.

### 1.2. Organization

In Section 2, we introduce existing studies on IoD environments. We provide a system model as well as an adversary model, fuzzy extractor, and PUF used in the proposed scheme in Section 3. Then, we show Akram et al.’s scheme in Section 4. Section 5 describes security vulnerabilities discovered in Akram et al.’s scheme. The proposed scheme is introduced in Section 6. Security analyses, i.e., BAN logic, RoR model, AVISPA, are shown in Section 7, and performance analyses, i.e., security features, communication, computation costs, are shown in Section 8. In Section 9, we conclude our paper and describe future works.

## 2. Related Works

Since the basic concept of IoD environments was introduced by Gharibi et al. [1], various authentication schemes have been proposed over the past few years. In 2018, Wazid et al. [11] proposed an authentication scheme to provide remote users with drone services based on three-factor technology. To apply lightweight communication services, Wazid et al. utilize hash function and exclusive-OR operators. However, their scheme cannot prevent privileged insider and impersonation attacks. In 2019, Teng et al. [12] analyzed security vulnerabilities, named “attacker mode”, which can happen in IoD environments. Thus, they proposed an authentication scheme utilizing the elliptic curve digital signature algorithm (ECDSA) to verify the legitimacy of identity signatures on drones. However, Teng et al.’s scheme was designed as an authentication scheme involving two-way authentication between drones based on ECC, which incurs a large computational overhead. Srinivas et al. [13] proposed a temporal credential-based authentication for IoD networks. Srinivas et al. argued that security and efficiency are the main requirements for the IoD environment, and a lightweight authentication protocol is essential to satisfy these requirements. In their scheme, the authors claimed that it can resist various security attacks such as a stolen mobile device, replay, MITM, ephemeral secret leakage (ESL), impersonation, password and/or biometric update, and remote drone capture attacks. In 2020, Ali et al. [14] pointed out that Srinivas et al.’s scheme [13] does not provide untraceability and resists stolen verifier attacks. To overcome that, Ali et al. suggested a lightweight authentication scheme for drones using symmetric key primitives and temporal credentials. Ever [15] suggested a framework for mobile sinks used in drones using bilinear pairing and ECC, which has a large computational cost. However, Ever’s protocol cannot provide user anonymity and untraceability [16]. In 2022, Wu et al. [17] proposed a drone communication scheme for 5G networks. They argued that several existing IoD protocols have high computation overheads because of using a public key infrastructure (PKI) mechanism. Therefore, they only utilized hash functions and exclusive-OR operators. In the same year, Tanveer et al. [18] proposed an authentication mechanism for IoD environments. They used an AES-CBC-256 cipher and ECC to ensure the anonymity of users. Although the above schemes [11,12,13,14,15,17,18] provide useful services such as healthcare, rescue, and traffic surveillance, they can suffer from physical attacks because each drone cannot protect security parameters from power analysis attacks.

To strengthen the authentication process and access control of drones, various PUF-based authentication schemes have been proposed. Alladi et al. [19] proposed a two-stage authentication protocol that divided drone hierarchies for smart drone networks. In Alladi et al.’s scheme, each drone equipped with PUF communicates with a ground station through a leader drone, reducing network overhead. Thus, the authors claimed their scheme does not require the storage of secret keys in drones, protecting it from impersonation, drone tampering, and MITM attacks. In the same years, Pu et al. [20] proposed an authentication protocol for drone environments using PUF and chaotic systems. The authors used the challenge–response pair of the PUF as the seed value of the chaotic system to jumble the message randomly. In 2021, Zhang et al. [21] suggested a three-party authentication scheme for IoD environments. In Zhang et al.’s scheme, the head drone manages member drones and mediates the communication between the ground station and member drones. The entire process of their scheme only uses hash functions and XOR operations. Moreover, the authors introduced PUF systems to prevent physical capture attacks.

In 2021, Akram et al. [4] suggested a scheme for secure and efficient drone access in IoD networks. The authors demonstrated that various security attacks, e.g., user, control center, and drone impersonation attacks, can be prevented in their scheme. However, our security analysis indicates that their scheme is vulnerable to DoS, session key disclosure, stolen-verifier, and drone impersonation attacks and cannot provide perfect forward secrecy.

We summarize the cryptographic techniques and the advantages and limitations of the existing related schemes [4,11,12,13,14,15,17,18,19,20,21] in Table 1. Although previous authentication schemes can provide convenient services to users, they still have high computational and communication overhead and security drawback problems. Therefore, we propose a secure drone-access scheme to improve these security flaws considering lightweight communication characteristics of IoD environments. The proposed scheme can provide stolen mobile device and drone impersonation attacks using biometric and PUF technologies, respectively. Moreover, the proposed scheme can support efficient communications using only hash functions and exclusive-OR operators.

## 3. Preliminaries

We present the system model and adversary model for IoD environments. Moreover, we introduce some relevant preliminaries to understand this paper.

### 3.1. System Model

**Figure 2:**
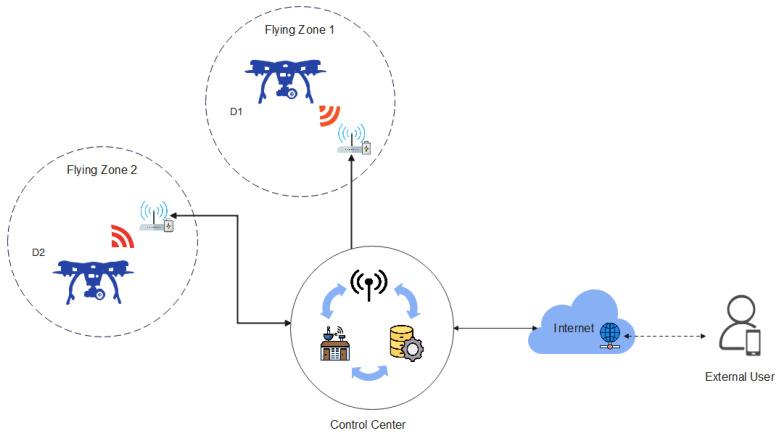
The general system model of IoD environments.

As shown in Figure 2, IoD environments consist of a control center, users and drones. According to the IoD environment model, various drones collect the data in their particular zones in a target field and transmit the data to the server. External users are required to connect to the server to obtain data from the deployed drones. For access, secure authentication is necessary between the user and drone via the control center. Subsequently, the user and drone pair share a session key and begin communication. The details of this process are as follows.

- Remote user ($U_{m}$): A remote user $U_{m}$ owns a mobile device to receive IoD services. To communicate with a drone $D_{n}$, $U_{m}$ must register with the control center. $U_{m}$ utilizes biometric technology in addition to identity and password to store sensitive information safely.
- Control center: The control center is a trusted third party with enough computation and storage capacities. Therefore, the control center perform a role as the system manager of IoD environments. Furthermore, the control center authenticates with both $U_{m}$ and $D_{n}$ information and helps $U_{m}$ to access the $D_{n}$. The control center generates secret keys for $U_{m}$ and $D_{n}$ against their identities.
- Drone ($D_{n}$): A drone $D_{n}$ collects the data in their particular flying zone and must be registered by the control center to communicate with $U_{m}$. Then, $D_{n}$ sends the data to =$U_{m}$ through the control center. Moreover, $D_{n}$ has restricted computation and storage capacities.

### 3.2. Adversary Model

We follow the widely used adversary model, named the “Dolev–Yao (DY) adversary model” [22,23]. Under the DY model, the entities involved in the IoD environments, i.e., $U_{m}$ and $D_{n}$, are not assumed to be trustworthy, and the communication of the channel is insecure. Therefore, an adversary A can modify or delete the transmitted messages and also can eavesdrop on the exchanged messages. Furthermore, drones move around in unattended hostile areas with collected sensor data. Thus, they are vulnerable to physical capture attacks [11,24], and the sensitive data stored in the drone can be extracted using the power analysis attacks.

### 3.3. Fuzzy Extractor

The fuzzy extractor [25] is widely accepted to verify the biometric authentication. A biometric key can be generated with a biometric template such as fingerprints, faces and irises. The fuzzy extractor is defined with the following two algorithms:

- $Gen\left(Bio_{m}\right)=\left(\alpha_{m},\beta_{m}\right)$: It is a probabilistic algorithm to generate a secret key $\alpha_{m}$. The user inputs biometric $Bio_{m}$, the output of this function is the secret parameter $\alpha_{m}$, and the public reproduction parameter $\beta_{m}$.
- $Rep\left(Bio_{m}^{*},\beta_{m}\right)=\left(\alpha_{m}\right)$: It is a deterministic algorithm to recreate the original $\alpha_{m}$. The function accepts a noisy user biometric $Bio_{m}^{*}$ and controls the noise using the public reproduction parameter $\beta_{m}$. Then, this algorithm reproduces the original biometric secret key $\alpha_{m}$.

### 3.4. Physical Unclonable Function

PUF is a physical circuit that maps a bit-string pair called “challenge–response pair” [6]. When an input challenge value is entered into the PUF circuit, it produces a value that isan arbitrary string of bits. In this paper, we use PUF to generate secret values instead of stringing them in the memory of the drone and obtain a stable response good enough for security using fuzzy extractors. The property of PUF is as below.

- The PUF is a physical microstructure of the device.
- It is extremely difficult or impossible to clone the PUF circuit.
- An unpredictable response value must be output.
- It is possible to evaluate and implement a PUF circuit easily.

## 4. Revisit of Akram et al.’s Scheme

Akram et al. [4] suggested a drone-access authentication protocol for surveillance tasks in a smart city. Akram et al.’s scheme is composed of the following phases: (1) user registration; (2) drone registration; (3) authentication and key agreement (AKA) phases. Table 2 shows the whole notation and description in their scheme.

### 4.1. Registration Phase

#### 4.1.1. Remote User Registration Phase

**Step 1:**  The user inputs their own $ID_{m}$, $PW_{m}$ and imprints $Bio_{m}$. Then, $U_{m}$ calculates $Gen\left(Bio_{m}\right)=\left(\alpha_{m},\beta_{m}\right)$ and sends $ID_{m}$ to the control center.
**Step 2:**  The control center calculates $SID_{m}=h(ID_{m}\left||s\right)$, $k_{m}=h(SID_{m}\left||MSK\right)$ and generates a random number $a_{m}$. After that, the control center computes $MID_{m}=Enc_{MSK}$$\left(SID_{m}||\alpha_{m}\right)$ and sends $\left\{k_{m},SID_{m},SID_{n}\right\}$ to $U_{m}$.
**Step 3:**  $U_{m}$ computes $\gamma_{m}=h(ID_{m}||PW_{m}\left|\right|\alpha_{m})⊕k_{m}$, $SID_{m}^{u}=h(ID_{m}||PW_{m})⊕SID_{m}$. Then, $U_{m}$ stores $\left\{\gamma_{m},SID_{m}^{u},SID_{n}\right\}$.

#### 4.1.2. Drone Registration Phase

**Step 1:**  $D_{n}$ selects $ID_{n}$ and sends it to the control center.
**Step 2:**  The control center computes $SID_{n}=h(ID_{n}\left||s\right)$, $k_{n}=h(SID_{n}\left||MSK\right)$ and stores $\left\{ID_{n},k_{n},SID_{n}\right\}$ in its database. Then, the control center sends $\left\{k_{n},SID_{n}\right\}$ to $D_{n}$.
**Step 3:**  When $D_{n}$ receives $\left\{k_{n},SID_{n}\right\}$, $D_{n}$ saves them in the memory.

### 4.2. AKA Phase

**Step 1:**  $U_{m}$ inputs $ID_{m}$, $PW_{m}$ and also imprints $Bio_{m}$. Then, $U_{m}$ computes $\alpha_{m}$$=Rep(Bio_{m},$$\beta_{m})$, $SID_{m}=SID_{m}^{u}⊕h(ID_{m}||PW_{m})$, $k_{m}=\gamma_{m}⊕h(ID_{m}||PW_{m}\left|\right|\alpha_{m})$. Afterward, $U_{m}$ generates $a_{1}$ and computes $A_{1}=h(SID_{m}||SID_{c}\left|\right|k_{m})⊕a_{1}$, $A_{2}=h(SID_{m}||SID_{c}\left|\right|k_{m}\left|\right|$$a_{1})⊕SID_{n}$ and $A_{3}=h(SID_{m}||SID_{n}||SID_{c}\left|\right|k_{m}\left|\right|a_{1})$. Finally, $U_{m}$ sends $\{MID_{m},$$A_{1},A_{2},A_{3}\}$ to the control center.
**Step 2:**  The control center retrieves $(SID_{m}\left|\right|\alpha_{m})=Dec_{MSK}\left(MID_{m}\right)$. Then, the control center computes $k_{m}=h(SID_{m}\left|\right|$MSK), $a_{1}^{*}=A_{1}⊕h(SID_{m}^{*}\left||SIDc|\right|k_{m}^{*})$ and $SID_{n}^{*}=A_{2}⊕h(SID_{m}^{*}||SID_{c}\left|\right|k_{m}^{*}\left|\right|a_{1}^{*})$, and verifies $k_{n}$ against $SID_{n}^{*}$. Then, the control center computes $A_{3}^{*}=h(SID_{m}^{*}||SID_{n}^{*}||SID_{c}\left|\right|k_{m}^{*}\left|\right|a_{1}^{*})$ and checks $A_{3}^{*}\overset{?}{=}A_{3}$. The control center generates $a_{2}$, $a_{m}^{new}$ and computes $MID_{m}^{new}=Enc_{MSK}(SID_{m}\left|\right|a_{m}^{new})$, $A_{4}=h(SID_{n}^{*}\left|\right|k_{n})⊕(a_{1}^{*}\left|\right|a_{2}||MID_{m}^{new})$, $A_{5}=h(SID_{n}^{*}||SID_{c}\left|\right|k_{n}\left|\right|a_{1}^{*})⊕SID_{m}^{*}$ and $A_{6}=h(SID_{m}^{*}||SID_{n}^{*}||SID_{c}\left|\right|k_{n}\left|\right|a_{1}^{*}\left|\right|a_{2})$. Finally, the control center sends $\{A_{4},A_{5}$, $A_{6}\}$ to the drone $D_{n}$.
**Step 3:**  $D_{n}$ computes $(a_{1}^{**}\left|\right|a_{2}^{*}||MID_{m}^{new})=A_{4}⊕h(SID_{n}\left|\right|k_{n})$, $SID_{m}^{**}=A_{5}⊕h(SID_{n}\left|\right|SID_{c}$$\left|\right|k_{n}\left|\right|a_{1}^{**})$ and $A_{6}^{*}=h(SID_{M}^{**}||SID_{n}\left|\right|$$SID_{c}\left|\right|k_{n}\left|\right|a_{1}^{**}\left|\right|a_{2}^{*})$. Then, $D_{n}$ checks $A_{6}^{*}\overset{?}{=}A_{6}$ and generates $a_{3}$. After that, $D_{n}$ computes $A_{7}=h(SID_{n}||SID_{m}^{**}\left|\right|a_{1}^{**})⊕(a_{2}\left|\right|a_{3}^{*}$$||MID_{m}^{new})$, $A_{8}=h(a_{1}^{**}\left|\right|a_{2}\left|\right|a_{3}^{*})$, $SK_{nm}=h(SID_{m}^{**}||SID_{n}||SID_{c}\left|\right|A_{8})$ and $A_{9}=h(SID_{m}^{**}||SID_{n}\left|\right|$$SID_{c}\left|\right|a_{2}\left|\right|a_{3}^{*}\left|\right|A_{8})$. Finally, $D_{n}$ sends $\{A_{7}$, $A_{9}\}$ to $U_{m}$.
**Step 4:**  The $U_{m}$ computes $\left(a_{2}^{*}|\right|$$a_{3}^{**}||MID_{m}^{new})=A_{7}⊕h(SID_{n}||SID_{m}\left|\right|a_{1})$, $A_{8}^{*}=h(a_{1}\left|\right|a_{2}^{*}$$\left|\right|a_{3}^{**})$ and $A_{9}^{*}=h(SID_{m}||SID_{n}||SID_{c}\left|\right|$$a_{2}^{*}\left|\right|a_{3}^{**}\left|\right|A_{8}^{*})$. Then, it validates $A_{9}^{*}\overset{?}{=}A_{9}$ and computes $SK_{nm}=h(SID_{m}^{**}||SID_{n}\left|\right|$$SID_{c}\left|\right|A_{8}^{*})$.

## 5. Cryptanalysis of Akram et al.’s Scheme

According to Section 3.2, an adversary A can obtain a $\{\gamma_{m},SID_{m}^{u},$$SID_{n}\}$ from legitimate user’s mobile device. Moreover, A can obtain $\left\{k_{n},SID_{n}\right\}$ from a captured drone using a power analysis attack. With this information, various security attacks, i.e., session key disclosure, drone impersonation, stolen-verifier, DoS attacks, and perfect forward secrecy, can be executed by A. The details are shown below.

### 5.1. Session Key Disclosure Attack

For A to generate a session key $SK_{nm}=h(SID_{m}||SID_{n}||SID_{c}\left|\right|A_{8})$, A has to obtain $SID_{m},SID_{n}$ and $A_{8}=h(a_{1}\left|\right|a_{2}\left|\right|a_{3})$. The procedures are as follows.

**Step 1:**  A computes $(a_{1}\left|\right|a_{2}||MID_{m}^{new})=A_{4}⊕h(SID_{n}\left|\right|$$k_{n})$, $SID_{m}=A_{5}⊕h(SID_{n}\left|\right|$$SID_{c}\left|\right|k_{n}\left|\right|a_{1})$, and $(a_{2}\left|\right|a_{3}||MID_{m}^{new})=A_{7}⊕h(SID_{n}||SID_{m}\left|\right|a_{1})$.
**Step 2:**  A calculates $SK_{nm}=h(SID_{m}||SID_{n}||SID_{c}\left|\right|A_{8})$.

Thus, Akram et al.’s scheme is insecure against session key disclosure attacks.

### 5.2. Drone Impersonation Attack

In this attack, we assume that A can capture drones $D_{n}$ physically and obtain the value $\left\{SID_{n},k_{n}\right\}$ stored in the memory of $D_{n}$. In order to be able to forward message $\left\{A_{7},A_{9}\right\}$ on behalf of legal $D_{n}$, then A has to calculate the value of $A_{7}=h(SID_{n}||SID_{m}\left|\right|a_{1})⊕(a_{2}\left|\right|a_{3}||MID_{m}^{new})$, $A_{9}=h(SID_{m}||SID_{n}||SID_{c}\left|\right|a_{2}\left|\right|a_{3}\left|\right|A_{8}).$A can compute the $A_{7}$ and $A_{9}$ through the following below:

**Step 1:**  The adversary A first intercepts $\left\{A_{4},A_{5},A_{6}\right\}$ transmitted by the public channel.
**Step 2:**  A can obtain $a_{1},a_{2}$, $MID_{m}^{new}$ by computing $(a_{1}\left|\right|a_{2}||MID_{m}^{new})=A_{4}⊕h(SID_{n}\left|\right|k_{n})$.
**Step 3:**  A can compute $SID_{m}$ through $SID_{m}=A_{5}⊕h(SID_{n}||SID_{c}\left|\right|k_{n}\left|\right|a_{1})$.
**Step 4:**  A generates random $a_{3}^{*}$ and computes $A_{8}^{*}=h(a_{1}\left|\right|a_{2}\left|\right|a_{3}^{*})$.
**Step 5:**  A can successfully compute $A_{7}^{*}=h(SID_{n}||SID_{m}\left|\right|$$a_{1})⊕(a_{2}\left|\right|a_{3}^{*}||MID_{m}^{new})$, $A_{9}^{*}=h(SID_{m}||SID_{n}||SID_{c}\left|\right|$$a_{2}\left|\right|a_{3}^{*}\left|\right|A_{8}^{*})$.

Therefore, Akram et al.’s scheme cannot resist drone impersonation attacks.

### 5.3. Stolen-Verifier Attack

When A obtains the table information $\left\{k_{n},SID_{n}\right\}$ of the control center, A can calculate $SK_{nm}=h(SID_{m}||SID_{n}||SID_{c}\left|\right|A_{8})$. The steps are the same as Section 5.1. Therefore, Akram et al.’s scheme is vulnerable to stolen-verifier attacks.

### 5.4. Perfect Forward Secrecy

Let us suppose that the control center’s long-term secret key MSK is compromised by the adversary A, and A has captured all the previously transmitted messages $MID_{m},A_{1},A_{2}$ and $A_{4}$ through the public channel. A can retrieve $SID_{m}$ through $(SID_{m}\left|\right|a_{m})=Dec_{MSK}$$\left(MID_{m}\right)$, compute $k_{m}=h(SID_{m}\left||MSK\right)$, $a_{1}=A_{1}⊕h(SID_{m}||SID_{c}\left|\right|k_{m})$, $SID_{n}=A_{2}⊕h(SID_{m}||SID_{c}\left|\right|k_{m}\left|\right|a_{1})$, and $k_{n}=h(SID_{n}\left||MSK\right)$. Furthermore, A can retrieve $a_{1}$ and $a_{2}$ through $(a_{1}\left|\right|a_{2}||MID_{m}^{new})=A_{4}⊕h(SID_{n}\left|\right|k_{n})$ and compute $A_{8}=h(a_{1}\left|\right|a_{2}\left|\right|a_{3})$. Finally, A computes the session key $SK_{nm}=h(SID_{m}||SID_{n}||SID_{c}\left|\right|A_{8})$. Thus, Akram et al.’s scheme does not provide perfect forward secrecy.

### 5.5. DoS Attack

In the AKA phase, the login process is not executed normally in the remote user ($U_{m}$) side. Afterward, the inputs $ID_{m}$, $PW_{m}$, and $Bio_{m}$, $U_{m}$ compute $\alpha_{m}$, $SID_{m}$, and $k_{m}$. Then, $U_{m}$ immediately generates a random nonce and computes an authentication request message $\left\{MID_{m},A_{1},A_{3}\right\}$. Therefore, the adversary A can send unlimited amounts of login authentication request messages to the control center if A obtains a stolen/lost mobile device of $U_{m}$ and inputs a randomly selected identity, password, and biometrics. These messages can threaten the load on the control center. Thus, Akram et al.’s scheme is vulnerable to DoS attacks.

### 5.6. Correctness

In the user registration phase, the control center calculates the value of $MID_{m}$. After that, the $MID_{m}$ is not transmitted to $U_{m}$, and $U_{m}$ cannot compute it because the $MID_{m}$ is masked with MSK, which is the control center’s secret key. However, in the AKA phase, $U_{m}$ sends the $MID_{m}$ to the control center as the first transmitted message. Thus, Akram et al.’s scheme has a correctness problem.

## 6. Proposed Scheme

**Figure 3:**
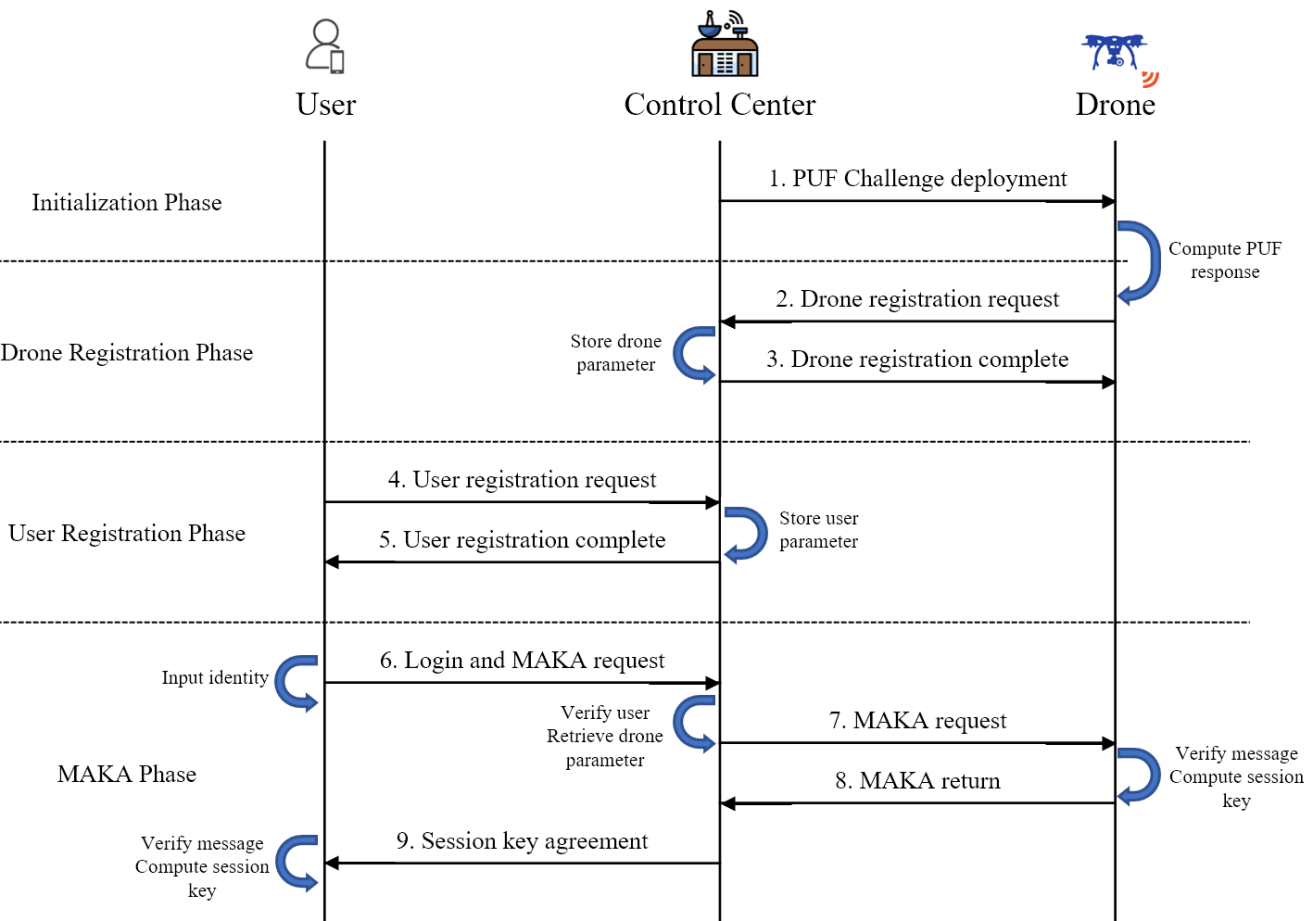
The overall flowchart of the proposed scheme.

The proposed scheme consists of the following phases: (1) initialization; (2) user registration; (3) drone registration; (4) MAKA. We show the flowchart of the proposed scheme in Figure 3. The proposed scheme is lightweight as it uses only the cryptographic one-way hash function and exclusive-OR operations, apart from the fuzzy extractor and PUF technique that is needed for verification at the user side and drone side, respectively.

### 6.1. Initialization Phase

**Figure 4:**
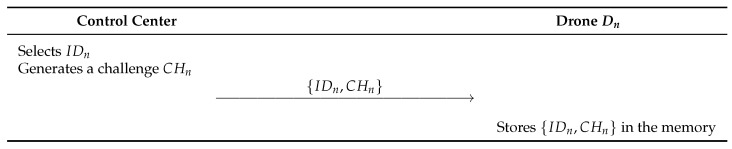
Initialization phase of the proposed scheme.

This phase describes that the control center selects an identity and a challenge for the drone $D_{n}$ before the registration phase. Detailed steps are illustrated in Figure 4. Additionally, this phase is performed via a secure channel.

**Step 1:**  The control center selects an identity $ID_{n}$ and a challenge $CH_{n}$ and sends $\left\{ID_{n},CH_{n}\right\}$ to the drone $D_{n}$.
**Step 2:**  The drone stores $\left\{ID_{n},CH_{n}\right\}$ in the memory.

### 6.2. Drone Registration Phase

**Figure 5:**
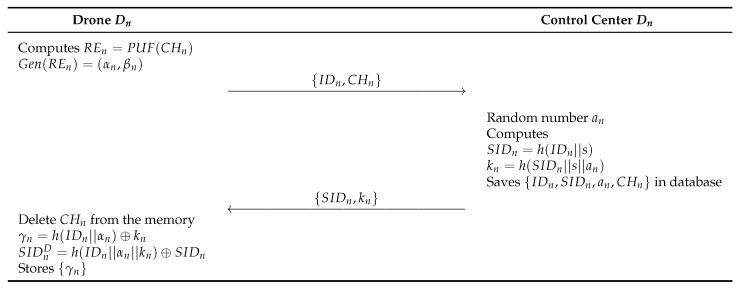
Drone registration phase of the proposed scheme.

In this phase, a drone $D_{n}$ is registered at the control center to its deployment in the IoD environments through a secure channel. Detailed steps are illustrated in Figure 5.

**Step 1:**  The drone $D_{n}$ retrieves the challenge $CH_{n}$ stored in the memory and computes $RE_{n}=PUF\left(CH_{n}\right)$, and $Gen\left(RE_{n}\right)=\left(\alpha_{n},\beta_{n}\right)$. After that, the $D_{n}$ sends $\left\{ID_{n},CH_{n}\right\}$ to the control center.
**Step 2:**  The control center generates a random number $a_{n}$ and computes $SID_{n}=h(ID_{n}\left||s\right)$, $k_{n}=h(SID_{n}\left||s|\right|a_{n})$, and saves $\left\{ID_{n},SID_{n},a_{n},CH_{n}\right\}$ in the database. Then, the control center sends $\left\{SID_{n},k_{n}\right\}$ to the $D_{n}$.
**Step 3:**  Finally, the $D_{n}$ deletes the $CH_{n}$ and computes $\gamma_{n}=h(ID_{n}\left|\right|\alpha_{n})⊕k_{n}$, $SID_{n}^{D}=h(ID_{n}\left|\right|\alpha_{n}\left|\right|k_{n})⊕SID_{n}$, and stores $\left\{\gamma_{n}\right\}$ in its memory.

### 6.3. User Registration Phase

**Figure 6:**
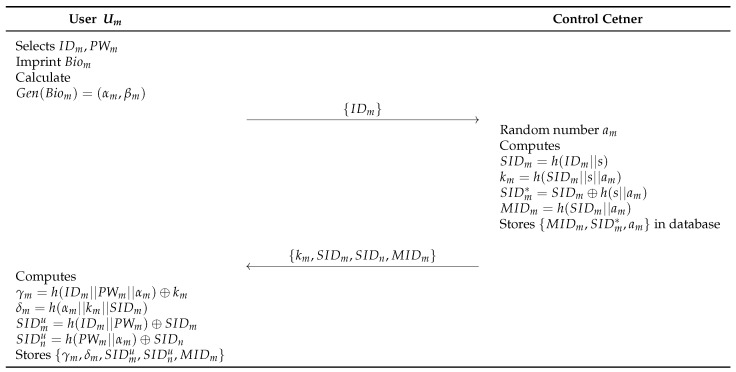
User registration phase of the proposed scheme.

In the user registration phase, a remote user $U_{m}$ has to register at the control center to access the real-time information from an accessed drone $D_{n}$ in IoD environments. This procure performs via a secure channel with the following steps. Figure 6 shows the details.

**Step 1:**  The user $U_{m}$ selects an identity $ID_{m}$, a password $PW_{m}$, and a biometric template $Bio_{m}$. After that, the mobile device calculates $Gen\left(Bio_{m}\right)=\left(\alpha_{m},\beta_{m}\right)$. The $U_{m}$ sends $\left\{ID_{m}\right\}$ to the control center.
**Step 2:**  The control center generates random number $a_{m}$ and computes $SID_{m}=h(ID_{m}$||s), $k_{m}=h(SID_{m}\left||s|\right|a_{m})$, $SID_{m}^{*}=SID_{m}⊕h(s||a_{m})$ and $MID_{m}=h(SID_{m}\left|\right|a_{m})$. Then, the control center stores $\left\{MID_{m},SID_{m}^{*},a_{m}\right\}$ in the database, and sends $\{k_{m},SID_{m}$, $SID_{n},MID_{m}\}$ to the $U_{m}$.
**Step 3:**  The $U_{m}$ computes $\gamma_{m}=h(ID_{m}||PW_{m}\left|\right|\alpha_{m})⊕k_{m}$, $\delta_{m}=h(\alpha_{m}\left|\right|k_{m}||SID_{m})$, $SID_{m}^{u}$$=h(ID_{m}||PW_{m})⊕SID_{m}$, and $SID_{n}^{u}=h(PW_{m}\left|\right|\alpha_{m})⊕SID_{n}$, and stores $\{\gamma_{m},\delta_{m},$$SID_{m}^{u},SID_{n}^{u},MID_{m}\}$ in the memory.

### 6.4. MAKA Phase

**Figure 7:**
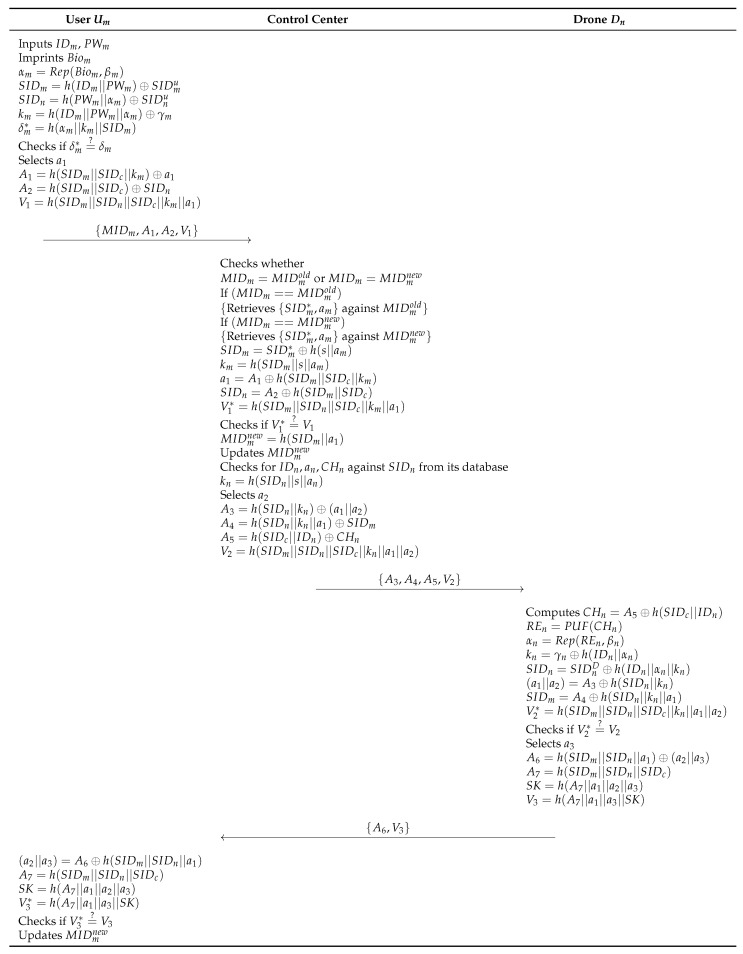
MAKA phase of the proposed scheme.

The following steps are performed among the $U_{m}$, the control center, and an accessed drone $D_{n}$ through a public channel. To establish a session key for secure communication among them, they need to perform the MAKA processes. Details are illustrated in Figure 7.

**Step 1:**  The $U_{m}$ inputs $ID_{m}$ and $PW_{m}$, and imprints $Bio_{m}$. After that, $U_{m}$ computes $\alpha_{m}=Rep\left(Bio_{m},\beta_{m}\right)$, $SID_{m}=h(ID_{m}||PW_{m})⊕SID_{m}^{u}$, $SID_{n}=h(PW_{m}\left|\right|\alpha_{m})⊕SID_{n}^{u}$, $k_{m}=h(ID_{m}||PW_{m}\left|\right|\alpha_{m})⊕\gamma_{m}$, and $\delta_{m}^{*}=h(\alpha_{m}\left|\right|k_{m}||SID_{m})$, and checks $\delta_{m}^{*}\overset{?}{=}\delta_{m}$. Then, the $U_{m}$ generates a random nonce $a_{1}$ and calculates $A_{1}=h(SID_{m}||SID_{c}\left|\right|k_{m})⊕a_{1}$, $A_{2}=h(SID_{m}||SID_{c})⊕SID_{n}$, and $V_{1}=h(SID_{m}\left|\right|SID_{n}$$||SID_{c}\left|\right|k_{m}\left|\right|a_{1})$. The $U_{m}$ sends $\left\{MID_{m},A_{1},A_{2},V_{1}\right\}$ to the control center.
**Step 2:**  The control center checks whether $MID_{m}=MID_{m}^{old}$ or $MID_{m}=MID_{m}^{new}$. If $\left(MID_{m}==MID_{m}^{old}\right)$ then, retrieves $\left\{SID_{m}^{*},a_{m}\right\}$ against $MID_{m}^{old}$, and if $\left(MID_{m}==MID_{m}^{new}\right)$, retrieves $\left\{SID_{m}^{*},a_{m}\right\}$ against $MID_{m}^{new}$. After that, the control center computes $SID_{m}=SID_{m}^{*}⊕h(s||a_{m})$, $k_{m}=h(SID_{m}\left||s|\right|a_{m})$, $a_{1}=A_{1}⊕h(SID_{m}||SID_{c}\left|\right|$$k_{m})$, $SID_{n}=A_{2}⊕h(SID_{m}||SID_{c})$, and $V_{1}^{*}=h(SID_{m}||SID_{n}||SID_{c}\left|\right|k_{m}\left|\right|a_{1})$. If $V_{1}^{*}\overset{?}{=}V_{1}$ is correct, the control center computes $MID_{m}^{new}=h(SID_{m}\left|\right|a_{1})$ and updates $MID_{m}^{new}$. Then, the control center checks for $ID_{n},a_{n},CH_{n}$ against $SID_{n}$ from its database and computes $k_{n}=h(SID_{n}\left||s|\right|a_{n})$. The control center calculates $A_{3}=h(SID_{n}\left|\right|k_{n})⊕(a_{1}\left|\right|a_{2})$, $A_{4}=h(SID_{n}\left|\right|k_{n}\left|\right|a_{1})⊕SID_{m}$, $A_{5}=h(SID_{c}||ID_{n})⊕CH_{n}$, and $V_{2}=h(SID_{m}||SID_{n}||SID_{c}\left|\right|k_{n}\left|\right|a_{1}\left|\right|a_{2})$ and sends $\left\{A_{3},A_{4},A_{5},V_{2}\right\}$ to the drone.
**Step 3:**  The drone $D_{n}$ computes $CH_{n}=A_{5}⊕h(SID_{c}||ID_{n})$, $RE_{n}=PUF\left(CH_{n}\right)$, $\alpha_{n}=Rep\left(RE_{n},\beta_{n}\right)$, $k_{n}=\gamma_{n}⊕h(ID_{n}\left|\right|\alpha_{n})$, $SID_{n}=SID_{n}^{D}⊕h(ID_{n}\left|\right|\alpha_{n}\left|\right|k_{n})$, $(a_{1}\left|\right|a_{2})=A_{3}⊕h(SID_{n}\left|\right|k_{n})$, $SID_{m}=A_{4}⊕h(SID_{n}\left|\right|k_{n}\left|\right|a_{1})$, and $V_{2}^{*}=h(SID_{m}||SID_{n}\left|\right|SID_{c}$$\left|\right|k_{n}\left|\right|a_{1}\left|\right|a_{2})$. If $V_{2}^{*}\overset{?}{=}V_{2}$ is correct, the $D_{n}$ generates a random nonce $a_{3}$, and calculates $A_{6}=h(SID_{m}||SID_{n}\left|\right|a_{1})⊕(a_{2}\left|\right|a_{3})$, $A_{7}=h(SID_{m}||SID_{n}||SID_{c})$, $SK=h(A_{7}||a_{1}||a_{2}||a_{3})$, and $V_{3}=h(A_{7}\left|\right|a_{1}\left|\right|a_{3}\left||SK\right)$. Then, the $D_{n}$ sends $\left\{A_{6},V_{3}\right\}$ to the $U_{m}$.
**Step 4:**  The $U_{m}$ computes $(a_{2}\left|\right|a_{3})=A_{6}⊕h(SID_{m}\left|\right|SID_{n}$$\left|\right|a_{1})$, $A_{7}=h(SID_{m}||SID_{n}\left|\right|$$SID_{c})$, $SK=h(A_{7}||a_{1}||a_{2}||a_{3})$, and $V_{3}^{*}=h(A_{7}\left|\right|a_{1}\left|\right|a_{3}\left||SK\right)$ and checks $V_{3}^{*}\overset{?}{=}V_{3}$. Then, the $U_{m}$ updates $MID_{m}^{new}$.

## 7. Security Analysis

To prove the security robustness of the proposed scheme, BAN logic, RoR model, and AVISPA simulation are used in this section. Using informal security analysis, we analyze the theoretical security of the proposed scheme.

### 7.1. BAN Logic

BAN logic [10] is a widely known formal proof used by many researchers to show mutual authentication of protocols [26,27,28]. Therefore, we apply the proposed scheme to BAN logic proof and verify mutual authentication. We introduce notations and descriptions for BAN logic in Table 3.

#### 7.1.1. Rules

In BAN logic, there are five logical rules: message meaning rule (MMR), nonce verification rule (NVR), jurisdiction rule (JR), belief rule (BR), and freshness rule (FR). Details are as follows.

**1.**  MMR:
  $$\frac{{\mathrm{PR}}_{1}\;|\equiv {\mathrm{PR}}_{1}\overset{KEY}{↔}{\mathrm{PR}}_{2},\;{\mathrm{PR}}_{1}⊲{\left(MSG_{1}\right)}_{KEY}}{{\mathrm{PR}}_{1}\left|\equiv {\mathrm{PR}}_{2}\right|∼MSG_{1}}$$
**2.**  NVR:
  $$\frac{{\mathrm{PR}}_{1}\left|\equiv \#\left(MSG_{1}\right),\;{\mathrm{PR}}_{1}\right|\equiv {\mathrm{PR}}_{2}\;|∼MSG_{1}}{{\mathrm{PR}}_{1}\left|\equiv {\mathrm{PR}}_{2}\right|\equiv MSG_{1}}$$
**3.**  JR:
  $$\frac{{\mathrm{PR}}_{1}|\equiv {\mathrm{PR}}_{2}⤇MSG_{1},\;{\mathrm{PR}}_{1}\left|\equiv {\mathrm{PR}}_{2}\right|\equiv MSG_{1}}{{\mathrm{PR}}_{1}\;|\equiv MSG_{1}}$$
**4.**  BR:
  $$\frac{{\mathrm{PR}}_{1}\;|\equiv \left(MSG_{1},MSG_{2}\right)}{{\mathrm{PR}}_{1}\;|\equiv MSG_{1}}$$
**5.**  FR:
  $$\frac{{\mathrm{PR}}_{1}\;|\equiv \#\left(MSG_{1}\right)}{{\mathrm{PR}}_{1}\;|\equiv \#\left(MSG_{1},MSG_{2}\right)}$$

#### 7.1.2. Goals

In the proposed scheme, there are four goals for the BAN logic. Let the user, control center, and drone be $U_{m}$, CC, and $D_{n}$, respectively.

**Goal 1:** 
$D_{n}|\equiv D_{n}\overset{SK}{↔}U_{m}$

**Goal 2:** 
$D_{n}\left|\equiv U_{m}\right|\equiv D_{n}\overset{SK}{↔}U_{m}$

**Goal 3:** 
$U_{m}|\equiv D_{n}\overset{SK}{↔}U_{m}$

**Goal 4:** 
$U_{m}\left|\equiv D_{n}\right|\equiv D_{n}\overset{SK}{↔}U_{m}$

#### 7.1.3. Idealized Forms

Three messages, i.e., $\left\{MID_{m},A_{1},A_{2},V_{1}\right\}$, $\left\{A_{3},A_{4},A_{5},V_{2}\right\}$, and $\left\{A_{6},V_{3}\right\}$, are transmitted via open channels in the proposed scheme. These messages are converted to idealized forms in BAN logic as below.

$Mes_{1}$

: $U_{m}\to CC:{\left\{a_{1},SID_{n}\right\}}_{SID_{m}}$

$Mes_{2}$

: $CC\to D_{n}:{\left\{a_{1},a_{2},SID_{m}\right\}}_{k_{n}}$

$Mes_{3}$

: $D_{n}\to U_{m}:{\left\{a_{2},a_{3}\right\}}_{SID_{m}}$

#### 7.1.4. Assumptions

We show the assumptions using in BAN logic as follows.

$AS_{1}$:
$CC|\equiv \#(a_{1})$

$AS_{2}$:
$D_{n}|\equiv \#\left(a_{2}\right)$

$AS_{3}$:
$U_{m}|\equiv \#\left(a_{3}\right)$

$AS_{4}$:
$D_{n}|\equiv U_{m}⤇\left(D_{n}\overset{SK}{↔}U_{m}\right)$

$AS_{5}$:
$U_{m}|\equiv D_{n}⤇\left(D_{n}\overset{SK}{↔}U_{m}\right)$

$AS_{6}$:
$CC|\equiv CC\overset{SID_{m}}{↔}U_{m}$

$AS_{7}$:
$D_{n}|\equiv CC\overset{k_{n}}{↔}D_{n}$

$AS_{8}$:
$U_{m}|\equiv D_{n}\overset{SID_{m}}{↔}U_{m}$

#### 7.1.5. BAN Logic Proof

**Step 1:**  We can obtain $RA_{1}$ from the message $Mes_{1}$.
$$RA_{1}:CC⊲{\left\{a_{1},SID_{n}\right\}}_{SID_{m}}$$
**Step 2:**  We can obtain $RA_{2}$ from the rule MMR using $RA_{1}$ and $AS_{6}$.
$$RA_{2}:CC|\equiv U_{m}|∼\left(a_{1},SID_{n}\right)$$
**Step 3:**  We can obtain $RA_{3}$ from the rule FR using $S_{3}$ and $AS_{1}$.
$$RA_{3}:CC|\equiv \#(a_{1},SID_{n})$$
**Step 4:**  We can obtain $RA_{4}$ from the rule NVR using $RA_{2}$ and $RA_{3}$.
$$RA_{4}:CC|\equiv U_{m}|\equiv \left(a_{1},SID_{n}\right)$$
**Step 5:**  We can obtain $RA_{5}$ from the message $Mes_{2}$.
$$RA_{5}:D_{n}⊲{\left\{a_{1},a_{2},SID_{m}\right\}}_{k_{n}}$$
**Step 6:**  We can obtain $RA_{6}$ from the MMR using $RA_{5}$ and $AS_{7}$.
$$RA_{6}:D_{n}\left|\equiv CC\right|∼\left(a_{1},a_{2},SID_{m}\right)$$
**Step 7:**  We can obtain $RA_{7}$ from the FR using $RA_{6}$ and $AS_{2}$.
$$RA_{7}:D_{n}|\equiv \#\left(a_{1},a_{2},SID_{m}\right)$$
**Step 8:**  We can obtain $RA_{8}$ from the NVR using $RA_{6}$ and $RA_{7}$.
$$RA_{8}:D_{n}\left|\equiv CC\right|\equiv \left(a_{1},a_{2},SID_{m}\right)$$
**Step 9:**  We can obtain $RA_{9}$ from the message $Mes_{3}$.
$$RA_{9}:U_{m}⊲{\left\{a_{2},a_{3}\right\}}_{SID_{m}}$$
**Step 10:**  We can obtain $RA_{10}$ from the MMR using $RA_{9}$ and $AS_{8}$.
$$RA_{10}:U_{m}\left|\equiv D_{n}\right|∼\left(a_{2},a_{3}\right)$$
**Step 11:**  We can obtain $RA_{11}$ from the NVR using $RA_{10}$ and $AS_{3}$.
$$S_{11}:U_{m}\left|\equiv D_{n}\right|\equiv \left(a_{2},a_{3}\right)$$
**Step 12:**  We can obtain $RA_{12}$ and $RA_{13}$ from $RA_{8}$ and $RA_{11}$. Therefore, $U_{m}$ and $D_{n}$ can compute the session key $SK=h(A_{7}||a_{1}||a_{2}||a_{3})$, where $A_{7}=h(SID_{m}||SID_{n}||SID_{c})$.
$$RA_{12}:D_{n}\left|\equiv U_{m}\right|\equiv \left(D_{n}\overset{SK}{↔}U_{m}\right)\;(Goal 2)$$
$$RA_{13}:U_{m}\left|\equiv D_{n}\right|\equiv \left(D_{n}\overset{SK}{↔}U_{m}\right)\;(Goal 4)$$
**Step 13:**  We can obtain $RA_{14}$ and $RA_{15}$ from the jurisdiction rule using $RA_{12}$ and $AS_{4}$, and $RA_{13}$ and $AS_{5}$, respectively.
$$RA_{14}:D_{n}|\equiv \left(D_{n}\overset{SK}{↔}U_{m}\right)\;(Goal 1)$$
$$RA_{15}:U_{n}|\equiv \left(D_{n}\overset{SK}{↔}U_{m}\right)\;(Goal 3)$$

### 7.2. RoR Model

The Real-or-Random model [9] is a formal proof analysis that proves the session key security of the protocol. Thus, we establish a premise for applying the proposed scheme to the RoR model. There are participants, adversaries and queries in our scheme. Participants are the entities that communicate with each other in the proposed scheme. Therefore, participants are as follows: $PAR_{U}^{i}$, $PAR_{C}^{j}$, and $PAR_{D}^{k}$, where *i*, *j*, and *k* are the instances of user, control center, and drone, respectively. The adversary in RoR model can modify, delete, and eavesdrop the exchanged messages. With this ability, the adversary can perform various queries such as Execute, CorruptDevice, Send, and Test. We describe the details of these queries as below.

- $Execute(PAR_{U}^{i},PAR_{C}^{j},PAR_{D}^{k})$: In this query, the adversary eavesdrop messages are transmitted via an open channel. Therefore, the adversary can obtain messages generated from $PAR_{U}^{i}$, $PAR_{C}^{j}$, and $PAR_{D}^{k}$. This query is a passive attack.
- $CorruptDevice(PAR_{U}^{i})$: In this query, the adversary can obtain secret parameters from $PAR_{U}^{i}$ using a power analysis attack. Therefore, the query *CorruptDevice* is an active attack.
- Send(PAR): In this query, the adversary can send messages to all participants $PAR_{U}^{i}$, $PAR_{C}^{j}$, and $PAR_{D}^{k}$. Furthermore, the adversary can obtain returned messages from these participants. Thus, this query is an active attack
- Test(PAR): Before starting the game, an unbiased coin UC is flipped in this query. The adversary obtains UC=1 when the session key is fresh. The adversary can also obtain UC=0 when the session key of the proposed scheme cannot guarantee freshness. If not, the adversary obtains a “null value” ⊥. To achieve a secure session key agreement, the adversary cannot discriminate between the session key and the random number.

#### Security Proof

**Theorem 1.**  *The adversary AD attempts to compute the session key $SK=h(A_{7}||a_{1}||a_{2}$$\left|\right|a_{3})$ in polynomial time. Therefore, we define the possibility that AD breaks the security of the session key as ${\mathrm{MA}}_{AD}\left(P\right)$. Moreover, we define that HA and PU are the range space of the function h(.) and PUF(.), respectively. The number of HA, PU, and Send queries are $qu_{ha}$, $qu_{pu}$, and $qu_{se}$, respectively. We define the secret biometric bits as $B_{m}$. At last, we define the Zipf’s parameter [29] as $C^{\prime }$ and $s^{\prime }$.*

$${\mathrm{MA}}_{AD}\left(P\right)\leq \frac{qu_{ha}^{2}}{\left|HA\right|}+\frac{qu_{pu}^{2}}{\left|PU\right|}+2max\left\{C^{\prime }qu_{se}^{s^{\prime }},\frac{qu_{se}}{2^{B_{m}}}\right\}$$

**Proof.**  The security proof in the proposed scheme is composed of five games $GA_{n}$(n=0,1,2,3,4). Before starting the game, we define $A_{GA_{n}}$ as the probability that AD wins the game and $AD[A_{GA_{k}}]$ as the advantage of $A_{GA_{k}}$. We follow the security proof according to [30,31,32].

$GA_{0}$:   In $GA_{0}$, the adversary selects a random bit *r*. Thus, we obtain the following equation.
  (1) $${\mathrm{MA}}_{AD}\left(P\right)=\left|2AD\left[A_{GA_{0}}\right]-1\right|$$
$GA_{1}$:   In $GA_{1}$, the adversary eavesdrops messages $\left\{MID_{m},A_{1},A_{2},V_{1}\right\}$, $\{A_{3},A_{4},A_{5},$$V_{2}\}$, and $\left\{A_{6},V_{3}\right\}$ using Execute query. Then, the adversary performs the Test query to obtain the session key $SK=h(A_{7}||a_{1}||a_{2}||a_{3})$. To compute SK, the adversary must obtain the random nonces $a_{1}$, $a_{2}$, and $a_{3}$. Moreover, $A_{7}$ is composed of $SID_{m}$, $SID_{n}$, and $SID_{c}$, where $SID_{m}$ is the secret parameter of user. Therefore, the adversary cannot calculate SK. Therefore, we can obtain the following equation.
  (2) $$|AD\left[A_{GA_{1}}\right]|=|AD\left[A_{GA_{0}}\right]|$$
$GA_{2}$:   In $GA_{2}$, the adversary utilizes Send and HA to attack the network. However, all of the parameters are masked in a cryptographic hash function that can prevent the hash collision problem. For this reason, the adversary cannot obtain the session key SK. According to the birthday paradox [33], we can obtain the following inequation.
  (3) $$|AD\left[A_{GA_{2}}\right]-AD\left[A_{GA_{1}}\right]|\leq \frac{qu_{ha}^{2}}{\left|HA\right|}$$
$GA_{3}$:   Similar to $GA_{2}$, the adversary utilizes queries Send and PU in this game. According to Section 3.4, the PUF is extremely difficult or impossible to clone. This means the adversary has no advantage in $GA_{3}$.
  (4) $$|AD\left[A_{GA_{3}}\right]-AD\left[A_{GA_{2}}\right]|\leq \frac{qu_{pu}^{2}}{\left|PU\right|}$$
$GA_{4}$:   This game is the final game in which the adversary extracts secret parameters $\left\{\gamma_{m},\delta_{m},SID_{m}^{u},SID_{n}^{u},MID_{m}\right\}$ from the device of the user using the query CorruptDevice. The adversary attempts to calculate SK from these parameters. However, each parameter consists of a password and the biometrics of a user, and this means that the adversary must guess the password and biometrics at the same time. Since this task is computationally infeasible, the adversary cannot compute SK. Therefore, we can obtain the following inequation using Zipf’s law [29].
  (5) $$|AD\left[A_{GA_{4}}\right]-AD\left[A_{GA_{2}}\right]|\leq max\left\{C^{\prime }qu_{se}^{s^{\prime }},\frac{qu_{se}}{2^{B_{m}}}\right\}$$

After the game, the adversary guesses the result bits *r*, and we can make the following equation.

(6) $$AD\left[A_{GA_{4}}\right]=\frac{1}{2}$$

We can calculate and obtain Equation (7) using (1) and (2).

(7) $$\frac{1}{2}{\mathrm{MA}}_{AD}\left(P\right)=|AD\left[A_{GA_{0}}\right]-\frac{1}{2}|=|AD\left[A_{GA_{1}}\right]-\frac{1}{2}|$$

Then, we can calculate and obtain Equation (8) from (6) and (7).

(8) $$\frac{1}{2}{\mathrm{MA}}_{AD}\left(P\right)=\left|AD\left[A_{GA_{1}}\right]-AD\left[A_{GA_{4}}\right]\right|$$

The result (9) can be obtained using the triangular inequality.

$$\frac{1}{2}{\mathrm{MA}}_{AD}\left(P\right)=\left|AD\left[A_{GA_{1}}\right]-AD\left[A_{GA_{4}}\right]\right|$$

$$\leq |AD\left[A_{GA_{1}}\right]-AD\left[A_{GA_{3}}\right]|$$

$$+|AD\left[A_{GA_{3}}\right]-AD\left[A_{GA_{4}}\right]|$$

$$\leq |AD\left[A_{GA_{1}}\right]-AD\left[A_{GA_{2}}\right]|$$

$$+|AD\left[A_{GA_{2}}\right]-AD\left[A_{GA_{3}}\right]|$$

$$+|AD\left[A_{GA_{3}}\right]-AD\left[A_{GA_{4}}\right]|$$

(9) $$\leq \frac{qu_{ha}^{2}}{2|HA|}+\frac{qu_{pu}^{2}}{2|PU|}+max\left\{C^{\prime }qu_{se}^{s^{\prime }},\frac{qu_{se}}{2^{B_{m}}}\right\}$$

After multiplying (9) by 2, we can obtain the required result inequation.

$${\mathrm{MA}}_{AD}\left(P\right)\leq \frac{qu_{ha}^{2}}{\left|HA\right|}+\frac{qu_{pu}^{2}}{\left|PU\right|}+2max\left\{C^{\prime }qu_{se}^{s^{\prime }},\frac{qu_{se}}{2^{B_{m}}}\right\}$$

Therefore, we can demonstrate that the proposed scheme can ensure the session key security by proving the Theorem 1. □

### 7.3. AVISPA Simulation

AVISPA [7,8] is a simulation tool that proves the security robustness of the proposed scheme against replay and MITM attacks. Therefore, various security protocols [23,34,35] are proved by using AVISPA. In this section, we explain the main data flow of AVISPA and show the simulation result.

Firstly, we need to write the proposed scheme as a programming language named “High-Level Protocol Specification Language (HLPSL)” in AVISPA. After writing in HLPSL code, the proposed scheme is converted to “Intermediate Format (IF)”. Then, the translator in AVISPA starts analyzing the IF through the four backends: “On-the-Fly Model Checker (OFMC)”, “Three Automata based on Automatic Approximations for Analysis of Security Protocol (TA4SP)”, “SAT-based Model Checker (SATMC)”, and “Constraint Logic-based Attack Searcher (CL-AtSe)”. Because OFMC and CL-AtSe only support an exclusive-OR operator, the proposed scheme is executed in these backends. The analyzed result is recorded and summarized in the “Output Format (OF)”. If there is a result of “SAFE” in OF, we can demonstrate that the proposed scheme can prevent replay and MITM attacks.

**Figure 8:**
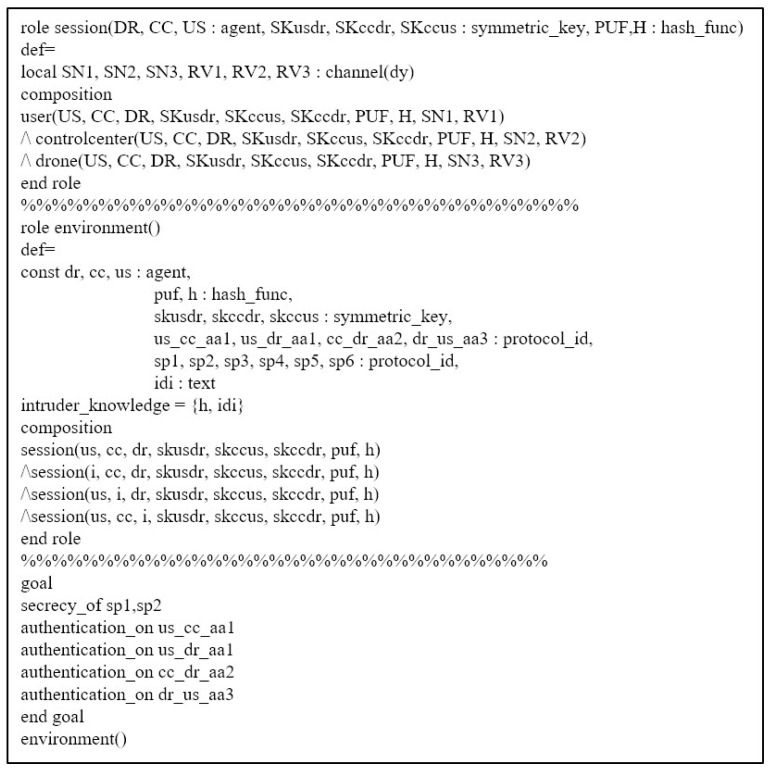
Session and environment roles written in HLPSL.

In AVISPA, we define roles to be suitable for the proposed scheme. Therefore, there are three roles in the proposed scheme: the user US, control center CC, and drone DR. Moreover, we show the session and environment roles in Figure 8.

**Figure 9:**
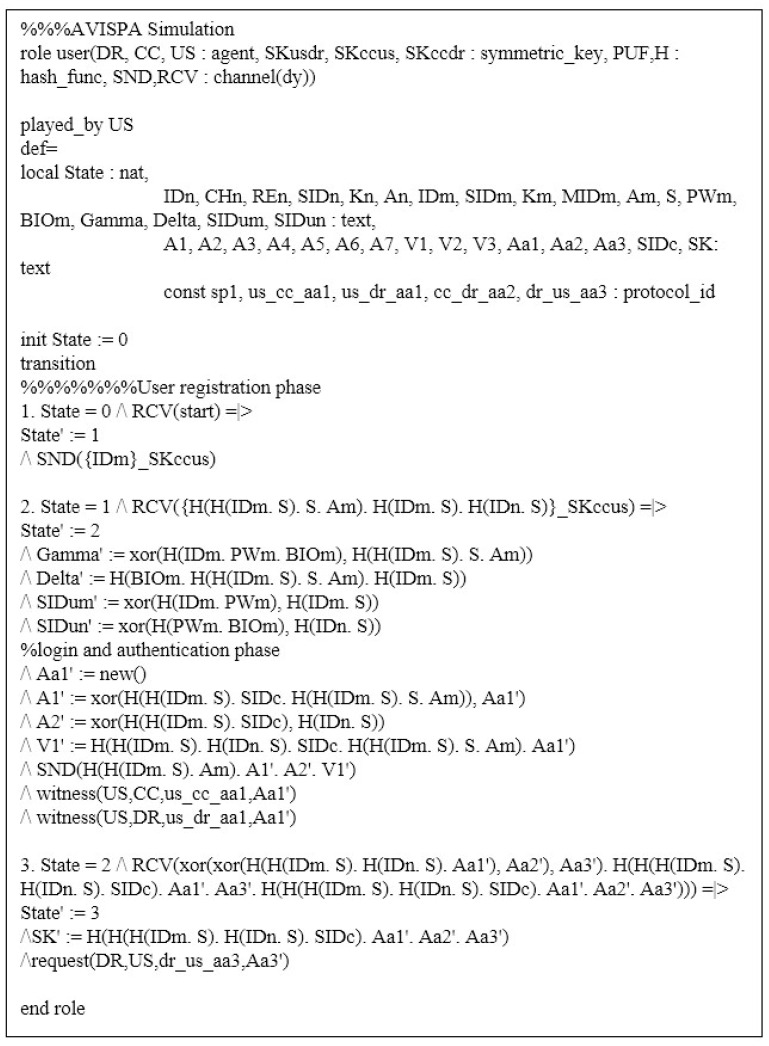
User role written in HLPSL.

Figure 9 shows the role of user US written in HLPSL code. State 1 is the user registration phase that US sends $\left\{ID_{m}\right\}$ to the CC through a secure channel. After receiving return message $\left\{k_{m},SID_{m},SID_{n},MID_{m}\right\}$ from CC, US computes and stores $\gamma_{m}$, $\delta_{m}$, $SID_{m}^{u}$, and $SID_{n}^{u}$ in state 2. Then, US computes a login request message $\left\{MID_{m},A_{1},A_{2},V_{1}\right\}$ to the CC. Note that $witness(US,CC,us\_cc\_aa1,Aa1^{\prime })$ and $witness(US,DR,us\_dr\_aa1,Aa1^{\prime })$ are functions to prove the freshness of random nonce $a_{1}$. Finally, US receives $\left\{A_{6},V_{3}\right\}$ from DR and computes the session key $SK=h(A_{7}||a_{1}||a_{2}||$$a_{3})$. The code $request(DR,US,dr\_us\_aa3,Aa3^{\prime })$ means the acceptance of freshness for $a_{3}$.

**Figure 10:**
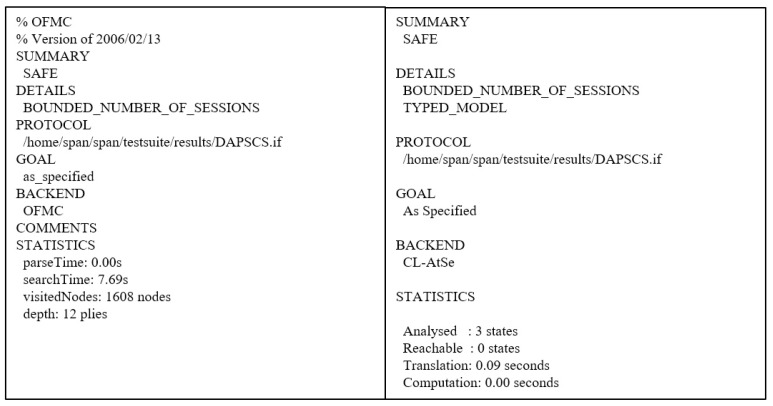
AVISPA result.

The AVISPA result is shown in Figure 10. As we mentioned before, we execute the proposed scheme in OFMC and CL-AtSe backends, and the summary of the result is “SAFE”. Therefore, we prove that the proposed scheme can prevent replay and MITM attacks.

### 7.4. Informal Security Analysis

We conduct an informal analysis of the proposed scheme to demonstrate the theoretical security robustness. Details are as below.

#### 7.4.1. Stolen/lost Mobile Device Attack

If an adversary A obtains a lost mobile device of $U_{m}$, it can extract secret parameters $\left\{\gamma_{m},\delta_{m},SID_{m}^{u},SID_{n}^{u},MID_{m}\right\}$ using power analysis attacks. However, all of secret parameters are masked in the identity $ID_{m}$, password $PW_{m}$, and biometrics $Bio_{m}$ information. Therefore, A must guess $ID_{m}$, $PW_{m}$, and $Bio_{m}$ at the same time and this process is not practical. Thus, the proposed scheme is secure against stolen/lost mobile device attacks.

#### 7.4.2. Offline Password-Guessing Attack

An adversary A can attempt an offline guessing attack using $\left\{MID_{m},A_{1},A_{2},V_{1}\right\}$, $\{A_{3}$, $A_{4}$$,A_{5},V_{2}\}$ and $\left\{A_{6},V_{3}\right\}$, and the extracted values $\{\gamma_{m},\delta_{m},$$SID_{m}^{u},SID_{n}^{u},MID_{m}\}$, $\left\{\gamma_{n}\right\}$ from mobile device and drone, respectively. Using a password dictionary, A can guess $PW_{A}^{*}$. However, A cannot know that $PW_{A}^{*}$ is valid or not. It is because $\delta_{m}$ is masked with biometric secret key $\alpha_{m}$. Therefore, the proposed scheme prevents offline password-guessing attacks.

#### 7.4.3. Impersonation Attack

(1) User impersonation attack: In this attack, an adversary A tries to disguise a legitimate user $U_{m}$. A has to make a valid login request message $\left\{MID_{m},A_{1},A_{2},V_{1}\right\}$. A can obtain $MID_{m}$ from the mobile device. However, without having the credentials $SID_{m},SID_{n}$, and $k_{m}$, it is a difficult task for A to calculate $MID_{m},A_{1},A_{2},V_{1}$. Thus, A cannot generate a valid login request message on behalf of $U_{m}$. Hence, the proposed scheme provides protection against user impersonation attacks.
(2) Control center impersonation attack: For this attack, let us suppose that A tries to send the message $\left\{A_{3},A_{4},A_{5},V_{2}\right\}$ to the $D_{n}$ on behalf of the CC. However, without having the credentials $SID_{m},SID_{n},k_{n},ID_{n}$, and random nonce $a_{1}$, it is computationally hard for A to make a valid message. Therefore, the proposed scheme is resilient against the CC impersonation attack.
(3) Drone impersonation attack: This attack is a disguise attack in which a malicious adversary A conceals its identity information and attempts to behave as $D_{n}$. To do this, A computes $CH_{A}^{*}=A_{3}⊕h(ID_{n}\left|\right|\gamma_{n})$. Since PUF(.) is a physical unclonable circuit, A cannot compute $RE_{n}$. Therefore, it is impossible to compute $\alpha_{n}=Rep\left(RE_{n},\beta_{n}\right)$, $SID_{n}=h(ID_{n}\left|\right|\alpha_{n})$, $k_{n}=\gamma_{n}⊕SID_{n}$, $(SID_{m}\left|\right|a_{1}\left|\right|a_{2})=A_{2}⊕h(SID_{n}||SID_{c}\left|\right|k_{n})$ to calculate $A_{4}=h(SID_{m}||SID_{n}\left|\right|a_{1})⊕(a_{2}\left|\right|a_{3})$. Thus, the proposed scheme can prevent drone impersonation attacks.

#### 7.4.4. Replay and MITM Attacks

In the proposed scheme, all messages are masked in random nonce $a_{1}$, $a_{2}$, and $a_{3}$ to maintain the freshness. Moreover, each participant, e.g., remote user, control center, drone, checks the validity of the message by calculating and checking $V_{1}^{*}$, $V_{2}^{*}$, and $V_{3}^{*}$. Therefore, the proposed scheme can prevent replay and MITM attacks.

#### 7.4.5. Physical and Cloning Attacks

For this attack, an adversary A intercepts a drone $D_{n}$ and extracts the secret parameters $\left\{\gamma_{n}\right\}$ from the memory. However, A cannot compute the session key $SK=h(A_{7}||a_{1}||a_{2}||a_{3})$ because each parameter in the message $\left\{A_{3},A_{4},A_{5},V_{2}\right\}$ is masked in the PUF technology, which has an unclonable property. Thus, A cannot obtain any advantages from $D_{n}$, and this means that the proposed scheme is secure against physical or cloning attacks.

#### 7.4.6. Privileged Insider Attack

In this attack, an adversary A is a privileged insider of the proposed system. Thus, A can obtain the registration request message $\left\{ID_{m}\right\}$ and secret parameters $\left\{\gamma_{m},\delta_{m},SID_{m}^{u},SID_{n}^{u},MID_{m}\right\}$ from the remote user $U_{m}$. However, without having $PW_{m}$ and biometric secret key $\alpha_{m}$ of $U_{m}$, deriving secret credentials $SID_{m}=h(ID_{m}||PW_{m})⊕SID_{m}^{u}$ and $k_{m}=h(ID_{m}||PW_{m}\left|\right|\alpha_{m})⊕\gamma_{m}$ is computationally infeasible. Thus, the proposed scheme prevents privileged insider attacks.

#### 7.4.7. Ephemeral Security Leakage Attack

To prevent this security attack, the proposed scheme must maintain security even if random numbers are leaked. Thus, A obtains $a_{1},a_{2},a_{3}$, which are used during the AKA phase. However, A cannot calculate $SID_{m},k_{m}$, and $k_{n}$ without knowing the secret key *s* to the control center. Additionally, A cannot obtain any advantages to impersonate as a legitimate user $U_{m}$. Thus, the proposed scheme prevents ephemeral secret leakage (ESL) attacks.

#### 7.4.8. Stolen-Verifier Attack

We can assume that an adversary A obtains table data $\left\{ID_{n},SID_{n},a_{n},CH_{n}\right\}$ and $\left\{MID_{m},SID_{m}^{*},a_{m}\right\}$ from the database of the control center and attempts to calculate the session key $SK=h(A_{7}||a_{1}||a_{2}||a_{3})$ or impersonate the control center. However, A cannot calculate the secret parameter $SID_{m},k_{m}$ and $k_{n}$ without the secret keys of the control center and also cannot obtain random number $a_{1},a_{2},a_{3}$. Thus, A cannot compute SK or impersonate the control center. This means that the proposed scheme is resilient to stolen-verifier attacks.

#### 7.4.9. User Anonymity and Untraceability

An adversary A cannot reveal the real identity $ID_{m}$ of a legitimate user because of a cryptographic one-way hash function h(.) masks $ID_{m}$ with the secret key of the control center. Therefore, the proposed scheme provides the user’s anonymity.

#### 7.4.10. Perfect Forward Secrecy

If the master key *s* of the control center is leaked to an adversary A, it can attempt to compute SK to attack the previous session. However, A cannot obtain the SK because $SK=h(A_{7}||a_{1}||a_{2}||a_{3})$ does not include *s*. Moreover, if master secret key *s* of the control center is compromised, A cannot obtain $SID_{m},SID_{n},a_{1},a_{2},a_{3}$ because A cannot compute $SID_{m}=h(ID_{m}\left||s\right)$ without the real identity of the $U_{m},SID_{n}=h(ID_{n}\left|\right|\alpha_{n})$ and without the secret key $\alpha_{n}$. Therefore, A does not obtain any advantages over SK. This means that the proposed scheme guarantees perfect forward secrecy.

#### 7.4.11. Mutual Authentication

In the MAKA phase, there are three messages $\left\{MID_{m},A_{1},A_{2},V_{1}\right\}$, $\left\{A_{3},A_{4},A_{5},V_{2}\right\}$, $\left\{A_{6},V_{3}\right\}$ transmitted via public channels. Thus, each participant checks the legitimacy of the other participants and messages using $V_{1}$, $V_{2}$, and $V_{3}$ in the proposed scheme. If this process is successful, we can ensure authentication. Thus, the proposed scheme guarantees mutual authentication.

#### 7.4.12. DoS Attack

If an adversary A tries to transmit $\left\{MID_{m},A_{1},A_{2},V_{1}\right\}$ to the control center as a replay message, A has to pass the login phase by verifying the values of $\delta_{m}=h(\alpha_{m}\left|\right|k_{m}||SID_{m})$. However, A cannot construct a valid $\delta_{m}$ because A cannot obtain $\alpha_{m},k_{m},SID_{m}$. Therefore, the replay message would not be sent to the control center. Thus, this proposed scheme can resist DoS attacks.

#### 7.4.13. Drone Capture Attack

If an adversary A captures a drone $D_{n}$ and obtains $\left\{\gamma_{n}\right\}$, A can try to threaten another legitimate drone $D_{n1}$. However, all of the drones are secure in PUF technology according to Section 7.4.5, and $\gamma_{n}=h(ID_{n}\left|\right|\alpha_{n})⊕k_{n}$ is an independent parameter. Therefore, the proposed scheme can prevent drone capture attacks.

#### 7.4.14. Session Key Disclosure Attack

To compute the session key $SK=h(A_{7}||a_{1}||a_{2}||a_{3})$, an adversary A has to obtain $SID_{m},SID_{n},a_{1},a_{2}$ and $a_{3}$. However, A cannot obtain any of these values because $SID_{m}$ and $SID_{n}$ are masked with secret key s and $a_{1},a_{2}$ and $a_{3}$ are random numbers that are temporarily used in a session. Therefore, the proposed scheme is secure against session key disclosure attacks.

## 8. Performance Analysis

We demonstrate the security features of the proposed scheme with a related sch- eme [4,14,18,21,24] in terms of “security functionalities”, “communication costs”, and “computation costs”.

### 8.1. Security Features Comparison

In order to provide visualized information, we offer comprehensive security properties of the proposed scheme and related schemes [4,14,17,18,21,24] in a table. As shown in Table 4, we consider various security functionalities and attacks, including “stolen smart card/mobile device”, “offline password guessing ”, “impersonation”, “replay”, “privileged-insider”, “physical and cloning”, “ESL”, “verification table leakage”, “user anonymity”, “perfect forward secrecy”, “mutual authentication”, “DoS”, “untraceability”, “device/drone capture”, and “correctness”. Thus, our scheme offers secure and functional features as compared to the related schemes [4,14,18,21,24].

### 8.2. Communication Costs Comparison

We demonstrate the comparison analysis for communication costs of the proposed scheme with the other related schemes [4,14,17,18,21,24]. We refer to [4] and assume that the bit lengths for the hash function, random number, identity, PUF challenge, ECC point, and enc-decryption are 256, random, 160, 32, 160, and 128 bits, respectively. Thus, during the MAKA process of our scheme, the exchanged messages $\left\{MID_{m},A_{1},A_{2},V_{1}\right\}$ require (256+256+256+256=1024 bits), the message $\left\{A_{3},A_{4},A_{5},V_{2}\right\}$ requires (256+256+256+256=1024 bits), and the message $\left\{A_{6},V_{3}\right\}$ requires (256+256=512 bits), respectively. Table 5 shows the total communication costs of the proposed scheme and the related schemes.

**Figure 11:**
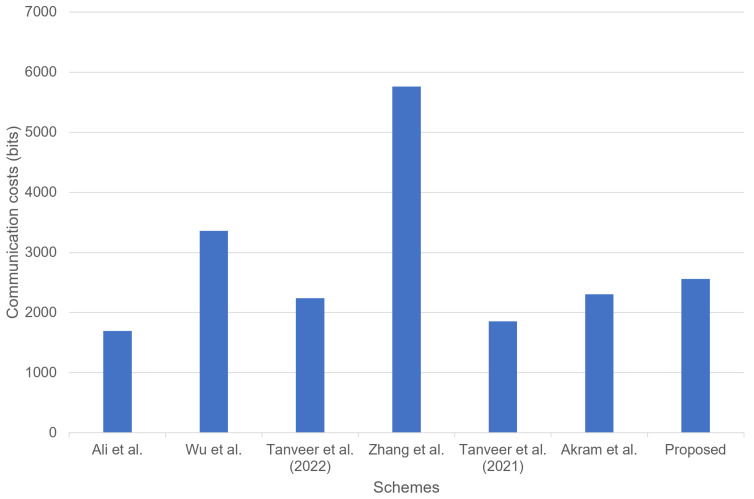
Communication costs comparison [4,14,17,18,21,24].

Although our scheme has slightly higher communication costs than Akram et al.’s scheme [4], we offer better security functionalities and efficient computation costs compared to the related schemes [14,17,18,21,24]. Figure 11 illustrates the total communication costs of the proposed scheme and the related schemes.

### 8.3. Computation Costs Comparison

We estimate the computation costs of the proposed scheme and [4,14,17,18,21,24] in the AKA phase. Referring to [18,21,24], we define that $T_{H}$, $T_{ECC}$, $T_{ENC}$, $T_{FE}$, $T_{AC}$, $T_{pm_{F}ourQ}$, $T_{M}$, and $T_{O}$ denote the hash function(≈0.029 ms), ECC multiplication(≈0.605 ms), enc-decryption time(≈0.036 ms), fuzzy extractor(≈0.605 ms), AEGIS(≈0.07 ms), FourQ point multiplication(≈1.199 ms), HMAC(≈0.053 ms), and BPV-online function(≈2.117 ms), respectively. Table 6 shows the total computation costs of the proposed scheme and the related schemes.

**Figure 12:**
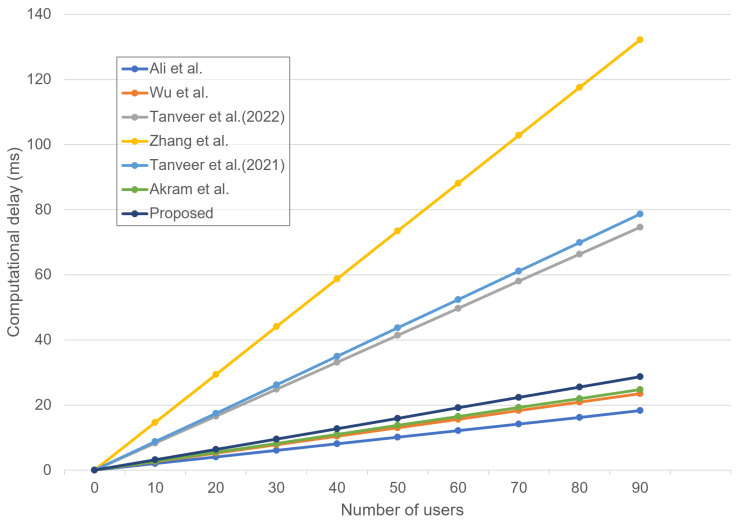
Computational delay at the control center with increasing the AKA requests [4,14,17,18,21,24].

Compared with the proposed scheme and Akram et al.’s scheme, the proposed scheme consumes more computation costs. However, the proposed scheme utilizes the fuzzy extractor and PUF technologies and, therefore, provides much higher security to the entire IoD network systems than [4]. Figure 12 illustrates that the computational cost (delay) increases at the control center with an increasing number of users.

## 9. Conclusions

In this study, we reviewed Akram et al.’s scheme, which was proposed for secure authentication between users and drones in IoD networks. In Akram et al.’s scheme, there are several security vulnerabilities, such as session key disclosure, drone impersonation, and stolen-verifier attacks. In addition, their scheme cannot ensure perfect forward secrecy and has correctness problems. To overcome the security flaws of their scheme and provide various functional features, we proposed a secure MAKA scheme using biometrics and PUF technologies. The proposed scheme can provide robustness to withstand various attacks, including session key disclosure, verification table leakage, impersonation, ESL, and privileged insider attacks. Moreover, the proposed scheme can achieve mutual authentication, perfect forward secrecy, and anonymity. To prove the session key security and mutual authentication, we analyzed the proposed scheme using an RoR model and BAN logic, respectively. Furthermore, we simulated the proposed scheme using AVISPA and showed that the proposed scheme is resilient against replay and MITM attacks. A comparative study of functionality features, efficiency, and security shows the effectiveness of the proposed scheme. Therefore, we can demonstrate that the proposed scheme has security robustness compared to existing user authentication protocols for IoD environments with reasonable computation and communication overheads. These characteristics show that the proposed scheme can provide users with high security reliability and high-speed communication in IoD environments. In future work, we intend to implement the proposed scheme in real environments using the mobile device as a user, a desktop as a server, and Raspberry PI 4 as a drone.

## Figures and Tables

**Table 1:** Cryptographic technologies and properties of the related schemes for IoD environments.

Schemes	Cryptographic Technologies	Advantages and Limitations
Wazid et al. [11]	* Hash functions * Fuzzy extractor	* Presented IoD environments and utilized biometrics information to ensure the security of remote users * Vulnerable to privileged insider and impersonation attacks
Teng et al. [12]	* ECDSA	* Defined security threats in IoD environments named “attacker mode” * Requires large computation overheads
Srinivas et al. [13]	* Hash functions * Fuzzy extractor	* Used temporal credentials for mutual authentication * Vulnerable to untraceability and stolen verifier attacks
Ali et al. [14]	* Hash functions * Fuzzy extractor * Symmetric key primitives	* Anonymous and lightweight security solution using temporal credentials and symmetric key primitives * Vulnerable to ESL, physical and cloning attacks
Ever et al. [15]	* Bilinear pairings * ECC	* Analyzed studies utilized UAVs as mobile sinks * Require high computation overheads * Cannot provide anonymity and untraceability
Wu et al. [17]	* Hash functions * Fuzzy extractor	* Proposed a drone-to-user authentication scheme for 5G networks * Vulnerable to physical attacks due to the stored parameters in UAV
Tanveer et al. [18]	* Hash functions * Fuzzy extractor * ECC * Symmetric key primitives	* Provides anonymous communication to users using AES and ECC * Vulnerable to physical attacks due to the stored parameters in UAV
Alladi et al. [19]	* PUF * Message authentication code * Symmetric key primitives	* Classified drones by layer and proposed PUF-based two-stage authentication protocol * Vulnerable to replay, insider, server spoofing, DoS attacks
Pu et al. [20]	* PUF * Chaotic system	* Used PUF and chaotic map technologies to generate random key * Vulnerable to physical attacks because of a stored challenge value in the memory of UAV
Zhang et al. [21]	* Hash functions * Fuzzy extractor * FourQ * Symmetric key primitives	* Proposed authentication scheme using FourQ and BPV pre-computation technologies * Require high computation and communication overheads * Cannot provide user anonymity
Akram et al. [4]	* Hash functions * Fuzzy extractor * Symmetric key primitives	* Provide privacy of location information to remote users and drones * Vulnerable to drone impersonation, stolen verifier, and DoS attacks, and have correctness problem

**Table 2:** Notations and descriptions.

Notation	Description
$ID_{m},ID_{n}$	Identity of the user and drone
$SID_{c},SID_{m},SID_{n}$	Pseudonym of the control center, user and drone
$Bio_{m}$	Biometric of the user
$k_{m},k_{n}$	Master private key of the user and drone
s,MSK	Secret keys of the control center
Rep(.)	Fuzzy biometric reproduction
Gen(.)	Fuzzy biometric generator
$a_{1},a_{2},a_{3}$	Random numbers
SK	Session key
h(.)	Hash function
||	Concatenation operator
⊕	Exclusive-OR operator

**Table 3:** Basic notations in BAN logic.

Notation	Description
${\mathrm{PR}}_{1},{\mathrm{PR}}_{2}$	Principals
$MSG_{1},MSG_{2}$	Statements
SK	Session key
${\mathrm{PR}}_{1}|\equiv MSG_{1}$	${\mathrm{PR}}_{1}$ **believes** $MSG_{1}$
${\mathrm{PR}}_{1}|∼MSG_{1}$	${\mathrm{PR}}_{1}$ once **said** $MSG_{1}$
${\mathrm{PR}}_{1}⤇MSG_{1}$	${\mathrm{PR}}_{1}$ **controls** $MSG_{1}$
${\mathrm{PR}}_{1}⊲MSG_{1}$	${\mathrm{PR}}_{1}$ **receives** $MSG_{1}$
$\#MSG_{1}$	$MSG_{1}$ is **fresh**
${\left(MSG_{1}\right)}_{KEY}$	$MSG_{1}$ is **encrypted** with KEY
${\mathrm{PR}}_{1}\overset{KEY}{↔}{\mathrm{PR}}_{2}$	${\mathrm{PR}}_{1}$ and ${\mathrm{PR}}_{2}$ have shared key KEY

**Table 4:** Security and functionality features (SFF) comparison.

SFF	[14]	[17]	[18]	[21]	[24]	[4]	Proposed
SP1	✓	✓	✓	✓	✓	✓	✓
SP2	✓	✓	✓	✓	✓	✓	✓
SP3	✓	✓	✓	✓	✓	✓	✓
SP4	✓	✓	✓	✓	✓	✓	✓
SP5	✓	✓	✓	✓	×	✓	✓
SP6	×	×	×	×	×	×	✓
SP7	×	✓	✓	✓	✓	✓	✓
SP8	✓	✓	✓	✓	×	×	✓
SP9	✓	✓	✓	✓	✓	✓	✓
SP10	×	✓	✓	✓	✓	✓	✓
SP11	✓	✓	✓	✓	✓	✓	✓
SP12	✓	✓	✓	✓	✓	×	✓
SP13	✓	✓	✓	✓	✓	✓	✓
SP14	✓	✓	✓	✓	✓	×	✓
SP15	✓	✓	✓	✓	✓	×	✓

Note: *SP*1: stolen smart card/mobile device attack; *SP*2: offline password guessing attack; *SP*3: impersonation attack; *SP*4: replay attack; *SP*5: privileged-insider attack; *SP*6: physical and cloning attack; *SP*7: ESL attack; *SP*8: stolen-verifier attack; *SP*9: user anonymity; *SP*10: perfect forward secrecy; *SP*11: mutual authentication; *SP*12: DoS attack; *SP*13: untraceability; *SP*14: device/drone capture attack; *SP*15: correctness; ✓: Provide or support SFF. ×: Do not provide or support SFF.

**Table 5:** Comparison study of communication costs.

Schemes	Total Costs	Number of Messages
Ali et al. [14]	1696 bits	3 messages
Wu et al. [17]	3360 bits	3 messages
Tanveer et al. [18]	2240 bits	3 messages
Zhang et al. [21]	5760 bits	4 messages
Tanveer et al. [24]	1856 bits	3 messages
Akram et al. [4]	2304 bits	3 messages
Proposed	2560 bits	3 messages

**Table 6:** Comparison study of computation costs.

Schemes	Remote User Side	Control Center Side	Drone Side	Total	Total Costs (s)
[14]	$10T_{H}+1T_{FE}$	$7T_{H}$	$7T_{H}$	$24T_{H}+1T_{FE}$	≈1.301 ms
[17]	$12T_{H}+1T_{FE}$	$9T_{H}$	$8T_{H}$	$29T_{H}+1T_{FE}$	≈1.446 ms
[18]	$9T_{H}+4T_{ENC}$ $+3T_{ECC}$	$4T_{H}+3T_{ENC}+1T_{ECC}$	$7T_{H}+2T_{ENC}$ $+2T_{ECC}$	$20T_{H}+9T_{ENC}+6T_{ECC}$	≈4.534 ms
[21]	$7T_{H}+3T_{pmFourQ}+$ $1T_{ENC}+1T_{O}+1T_{M}$	$5T_{H}+1T_{pmFourQ}$ $+2T_{ENC}+1T_{M}$	$4T_{H}+1T_{pmFourQ}$ $+1T_{ENC}+1T_{O}$	$16T_{H}+5T_{pmFourQ}$ $+4T_{ENC}+2T_{O}+2T_{M}$	≈10.943 ms
[24]	$6T_{H}+3T_{AC}$ $+3T_{ECC}+1T_{FE}$	$2T_{H}+1T_{ECC}+3T_{AC}$	$3T_{H}+2T_{ECC}+2T_{AC}$	$11T_{H}+6T_{ECC}$ $+8T_{AC}+1T_{FE}$	≈5.114 ms
[4]	$9T_{H}$	$7T_{H}+2T_{ENC}$	$7T_{H}$	$23T_{H}+2T_{ENC}$	≈0.739 ms
Ours	$11T_{H}+1T_{FE}$	$11T_{H}$	$10T_{H}+1T_{FE}$	$32T_{H}+2T_{FE}$	≈2.138 ms

## Data Availability

Not applicable.
